# Cytogenetic analysis of spontaneously discharged products of conception by array-based comparative genomic hybridization

**DOI:** 10.1186/s40064-016-2594-6

**Published:** 2016-06-24

**Authors:** Nobuaki Ozawa, Haruhiko Sago, Kentaro Matsuoka, Tetsuo Maruyama, Ohsuke Migita, Yoshinori Aizu, Johji Inazawa

**Affiliations:** Center of Maternal-Fetal, Neonatal and Reproductive Medicine, National Center for Child Health and Development, 2-10-1 Okura, Setagaya-ku, Tokyo, 157-8535 Japan; Department of Pathology, National Center for Child Health and Development, Tokyo, Japan; Department of Obstetrics and Gynecology, Keio University School of Medicine, Tokyo, Japan; Department of Clinical Genetics and Molecular Medicine, National Center for Child Health and Development, Tokyo, Japan; Division of Advanced Technology and Development, BML, Inc., Kawagoe, Japan; Department of Molecular Cytogenetics, Medical Research Institute, Tokyo Medical and Dental University, Tokyo, Japan; Bioresource Research Center, Tokyo Medical and Dental University, Tokyo, Japan; Department of Pathology, Kitasato Institute Hospital, Tokyo, Japan; Department of Clinical Genetics, St. Marianna University School of Medicine, Kawasaki, Japan

**Keywords:** Products of conception (POC), Recurrent pregnancy loss (RPL), Chromosomal abnormality, Array-based comparative genomic hybridization (array-CGH), Short tandem repeat (STR)

## Abstract

**Background:**

Cytogenetic analysis of products of conception (POC) is essential for the management of recurrent pregnancy loss (RPL), but the currently-performed G-banding method is not necessarily applicable to spontaneously discharged POC because of poor quality for culture. We analyzed the karyotypes of 15 spontaneously discharged POC by array-based comparative genomic hybridization (array-CGH).

**Results:**

All specimens were successfully analyzed and 10 cases had abnormal results: gain in copy number (n = 7) and loss in copy number (n = 3). Most of them were estimated to be whole chromosome aneuploidy, whereas one case was compatible with microdeletion. Two cases were suspected to be male diploid contaminated by maternal DNA or triploid because of the unsatisfactory signal patterns on X/Y chromosomes. Two of three cases with normal female DNA pattern were identified to be contaminated with maternal DNA by the additional analysis of short tandem repeats.

**Conclusions:**

Given the potential to analyze non-viable POC specimens, array-CGH is a feasible cytogenetic tool for women, in particular, with a history of RPL who desire non-surgical or expectant management of miscarriages and/or a thorough investigation on the cause for recurrent miscarriage, although it needs to take into account high incidence of maternal contamination in spontaneously discharged POC.

**Electronic supplementary material:**

The online version of this article (doi:10.1186/s40064-016-2594-6) contains supplementary material, which is available to authorized users.

## Background

Spontaneous miscarriage is the most common complication in early pregnancy, occurring at the frequency of 10–15 % of all clinically recognized pregnancies. Approximately 50 % of miscarriages are caused by chromosomal abnormalities of conception, such as autosomal trisomy, polyploidy, monosomy X, and structural abnormalities (Stephenson and Kutteh 2007; van den Berg et al. 2012). Most of these abnormalities occur sporadically and become crucial obstacle for women hoping for a child. At present, cytogenetic analysis of spontaneously aborted products of conception (POC) is not routinely performed. For women with recurrent pregnancy loss (RPL), however, cytogenetic analysis of POC is practically essential for the evaluation of currently-performed treatments and the management of subsequent pregnancies (Stephenson et al. 2002; Brezina and Kutteh 2014). In case of structural abnormality inherited from either parent, preimplantation genetic diagnosis is recommended to avoid another miscarriage. Repeated aneuploidy results could be plausible indication of preimplantation genetic screening in future (Chang et al. 2011; Dahdouh et al. 2015).

Cytogenetic analysis of POC is traditionally performed by G-banding method on metaphase chromosome preparations derived from cultured chorionic villi. This standard method requires a sterile, viable specimen for tissue culture; therefore, scheduled dilation and curettage (D&C) is usually needed. Indeed, the success rate of cytogenetic analysis is significantly lower in the specimens collected following spontaneous passage of pregnant tissue when compared with those obtained by D&C (Stephenson et al. 2002). However, a large percentage of women suffering from incomplete miscarriages tend to choose expectant management (Luise et al. 2002). In addition, spontaneous passage frequently occurs prior to the scheduled day even in women electing D&C. Therefore, it is necessary to establish a cytogenetic analysis for spontaneously discharged POC.

Array-based comparative genomic hybridization (array-CGH) is a powerful cytogenetic tool that enables us to perform the genome-wide and high-resolution analysis of DNA copy number changes (Inazawa et al. 2004; Hayashi et al. 2011). In recent years, this technique has been utilized for analysis of POC and shown to detect more cytogenetic abnormalities than G-banding analysis, although it is uncertain whether some of them cause miscarriage (van den Berg et al. 2012; Dhillon et al. 2013). In addition to its high detection ability, array-CGH has an advantage in that it does not need to culture cells. In the present study, we assessed the feasibility of array-CGH for the cytogenetic analysis of spontaneously discharged POC.

## Results

**Fig. 1:**
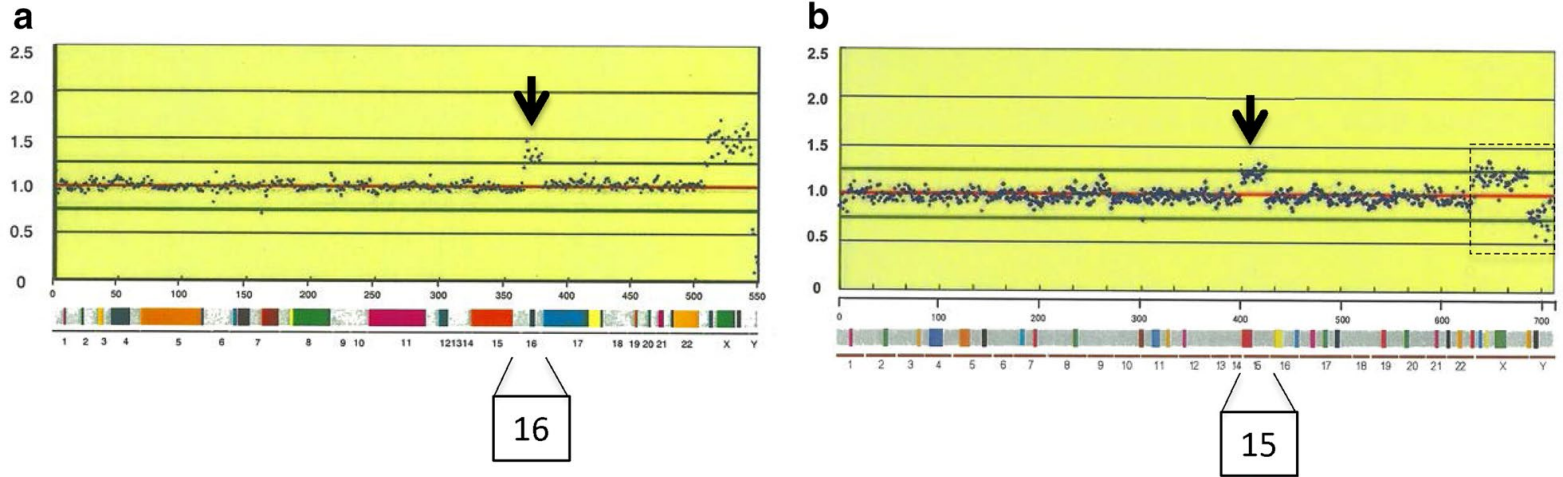
GDA results (Cases 6 and 11). The *x-axis* indicates array spots of BAC clones ordered from chromosomes 1–22, X and Y. The *y-axis* shows the fluorescence ratio of differently labeled sample/control DNA. The *color bars* indicate the regions of genetic diseases included in the respective GDA. **a** In Case 6, copy-number gain was recognized on all clones of chromosome 16 and X/Y signal patterns indicated that the case has the reverse gender to control. Accordingly, this case was estimated as a female with trisomy 16. **b** Case 11 was estimated as trisomy 15 with unsatisfactory signals on X/Y regions as indicated by a *dotted line box*. Each *arrow* indicates duplication signals on the corresponding chromosomal region

Fifteen specimens were collected from 13 women with missed abortion after ethical approval was obtained in 2007. The average maternal age at miscarriage was 35.7 years old, and the average week of gestation adjusted based on sonographic measurements was 5.9. All women had suffered from recurrent pregnancy loss before participation in this study except Cases 6 and 9. Two women had undergone miscarriage twice during this study and provided both miscarriage specimens (Table 1). All specimens were successfully analyzed by array-CGH using BAC (bacterial artificial chromosome)-based targeting array, designated Genome Disorder Array (GDA). 10 of 15 specimens (66.7 %) had copy number changes, consisting of gain in copy number (n = 7) and loss in copy number (n = 3) (Table 1) (Additional file 1: Figure S1). Gains or losses spreading on almost all BAC clones of a chromosome were interpreted as whole chromosome aneuploidy; although the genetic information outside the BAC clone regions was not available. The copy number changes restricted to a segmental region of a chromosome indicated a partial imbalance. In the present study, the majority of the specimens with copy number changes were estimated to have autosomal complete trisomy or monosomy. For instance, Case 6 was estimated as a female with trisomy 16 (Fig. 1a). Only one case (Case 2) showed microdeletion (1p36 loss), although the specimen was not available for confirmatory testing by FISH (fluorescence in situ hybridization) analysis. This microdeletion was less likely due to inheritance from either parent but more likely to be a de novo change because both parents had normal G-banding karyotypes and normal GDA findings (Additional file 2: Figure S2).

**Table 1:** Results of GDA on spontaneously discharged POC

Case	Maternal age(years)	Gestational weeks	Position of copy-number changes		Estimated karyotype (gender^a^)
			Gain	Loss	
1	37	6	22q11.1q11.22, 22q13.31	–	Trisomy 22 (F)
2	33	5	–	1p36.33p36.32	1p36 deletion (M)
3	38	5	–	–	Normal (F)
4	37	6	–	–	Normal (F)
5	37	6	11p13p12, 11q25	–	Trisomy 11 (M)
6	36	5	16p13.3, 16q24.3	–	Trisomy 16 (F)
7	33	6	–	21q22.13q22.2, 21q22.3	Monosomy 21 (M)
8	36	5	–	–	Normal (F)
9	37	5	22q11.1q11.21, 22q13.31q13.32	–	Trisomy 22 (F)
10	36	5	2p25.3, 2p11.2, 2q11.1q11.2, 2q22.3, 2q37.3	–	Trisomy 2 (M)
11	39	5	15q11.2q12, 15q24.1, 15q26.3	–	Trisomy 15 (M)^b^
12	40	6	–	21q11.2, 21q21.3, 21q22.13q22.2, 21q22.3	Monosomy 21 (M)
13	39	8	15q11.2q13.1, 15q24.1q24.2, 15q26.3	–	Trisomy 15 (M)
14	29	9	–	–	Normal (M)
15	28	7	–	–	Normal (M)^b^

^a^Estimated gender of POC; M = male, F = female. ^b^Cases 11 and 15 showed unsatisfactory signals on X/Y regions, suggesting to be mosaicism of XX and XY resulted from contamination with maternal DNA or triploidy as 70,XXY, + 15, 69,XXY, respectively. Two women had undergone miscarriage twice during this study and provided both miscarriage specimens: Cases 1/5 and Cases 2/7. Cases 1–6 were analyzed by GDA Ver. 2 (550BACs), Cases 7–9 by GDA Ver. 3 (660BACs) and Cases 10–15 by GD-700 (712BACs)

**Fig. 2:**
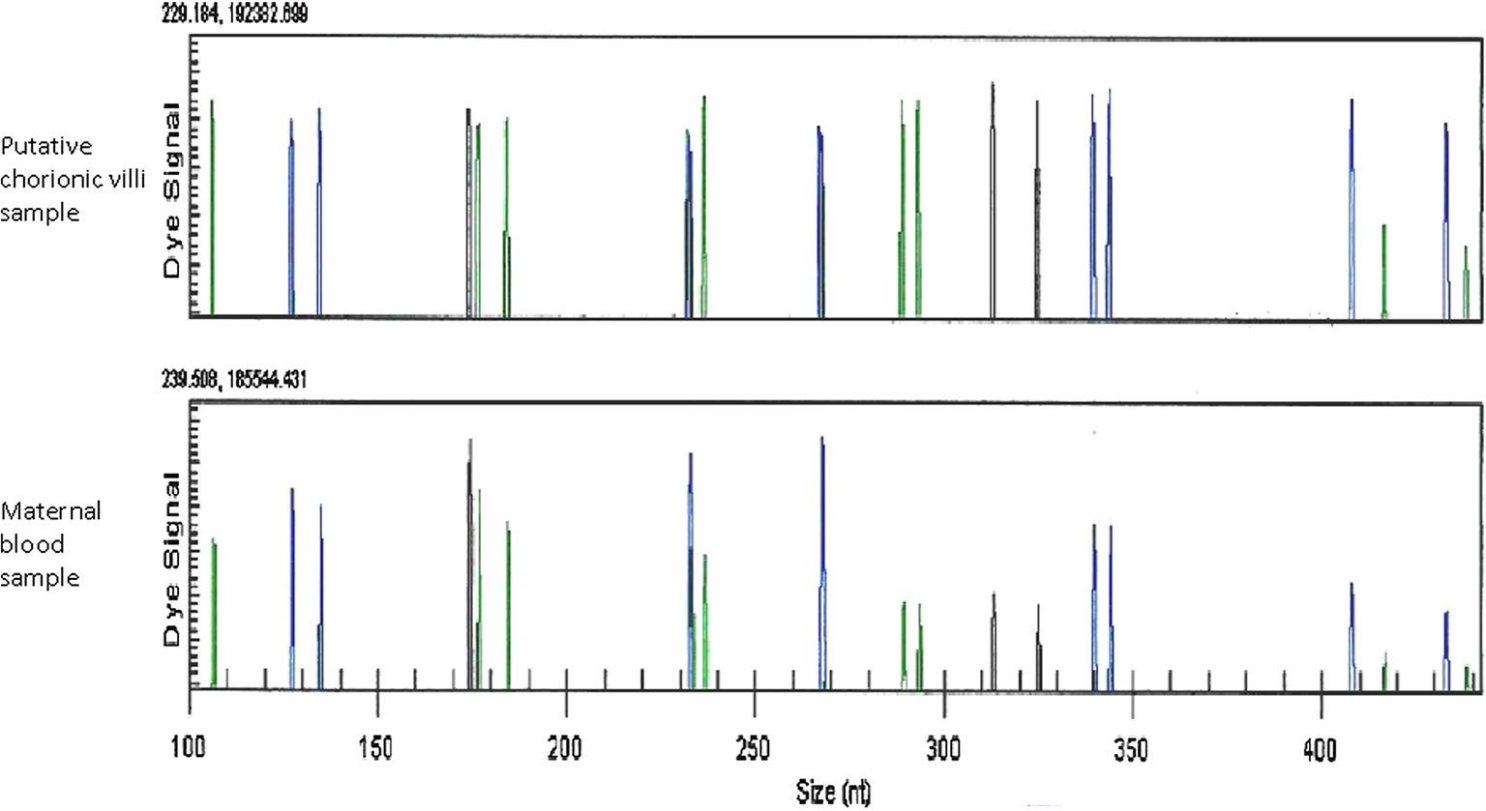
STR analysis result (Case 3). PCR products were visualized with the CEQ 8000 (Beckman Coulter). Both DNA samples from putative chorionic villi and maternal blood proved to have the same polymorphism patterns by STR analysis

In Cases 11 and 15, the signals on chromosomes X/Y did not show definite patterns as normal diploid, somewhat deviating from normal male or female pattern, accompanied with slightly decreased duplication signals on chromosome 15 in Case 11 (Fig. 1b). Two possibilities were considered as the cause: one possibility is that fetal male DNA was contaminated with maternal DNA, the other is that fetus was triploid as 70,XXY,+15, 69,XXY, respectively. In addition, short tandem repeat (STR) analysis revealed that both DNA extracted from putative chorionic villi and maternal blood had the same polymorphism patterns in two cases with normal female GDA results (Cases 3 and 4) (Fig. 2), suggesting that these specimens had little DNA from chorionic villi. Maternal blood DNA was not available in Case 8, although single diploid pattern was recognized by STR analysis.

## Discussion

This study was conducted to explore the utility of array-CGH supplemented with STR analysis for the cytogenetic analysis of POC collected following spontaneous discharge at home. To date, various genetic methods other than the conventional G-banding karyotyping have been proposed for the cytogenetic analysis of POC, including FISH and DNA-based analysis such as array-CGH, quantitative fluorescent polymerase chain reaction (QF-PCR) and multiplex ligation-dependent probe amplification (MLPA) (van den Berg et al. 2012; Kooper et al. 2014; Kim et al. 2015). Each of these methods, however, has some drawbacks and is insufficient when used in isolation. In the conventional G-banding method, a relatively high rate of culture failure, overgrowth of maternal cells, and the limited banding resolution are major shortcomings (van den Berg et al. 2012). Culture failure may frequently occur in spontaneously discharged specimens because of poor viability. On the other hand, DNA-based analysis is possible even in non-viable specimens if high quality DNA is extracted. In the present study, array-CGH analysis was successful in all cases, and the results of array-CGH findings were informed to the study participants for subsequent pregnancy management. Also, array-CGH analysis enables us to detect submicroscopic imbalances, not detectable by G-banding analysis (van den Berg et al. 2012; Dhillon et al. 2013; Viaggi et al. 2013; Zhou et al. 2016). Indeed, the terminal deletion of chromosome 1p (1p36) was identified in one of the present cases. This deletion results in a distinct postnatal phenotype with neurodevelopmental delay (Shapira et al. 1997), although it is unclear whether it caused the miscarriage.

Maternal cell contamination is a major troublesome obstacle to cytogenetic analysis of POC. In an earlier study, about 30 % of the first trimester miscarriage specimens with 46,XX karyotypes, diagnosed by conventional karyotyping, proved to have male karyotypes by additional polymerase chain reaction assay or FISH analysis (Bell et al. 1999). In order to minimize maternal contamination, it is extremely important to completely separate chorionic villi from maternal tissues (Lathi and Milki 2002). In spontaneously discharged POC, however, accurate identification of chorionic villi is often difficult due to extensive tissue degeneration. Indeed, two of 15 miscarriage specimens were suspected to be contaminated with maternal DNA by GDA analysis and the additional STR analysis revealed that two of three normal female cases contained mostly maternal DNA. Thus, the confirmatory assay should be additionally performed to evaluate maternal contamination, especially in the specimens with normal female array results. DNA extracted for array-CGH is also applicable for STR analysis.

Although array-CGH has several advantages compared to the conventional G-banding analysis, it is more costly and incapable of finding some polyploidies such as 69,XXX, 92,XXXX, 92,XXYY and balanced translocations (van den Berg et al. 2012; Gao et al. 2012; Dhillon et al. 2013). Polyploidies cause about 5–10 % of all first-trimester miscarriage (van den Berg et al. 2012). Balanced translocations are not the cause of miscarriage, but the findings suggest that either parent could be a balanced carrier and the results of parental karyotyping become useful information for future pregnancies. Therefore, the traditional G-banding analysis should be primarily performed for the specimens collected by D&C in the setting of RPL. If, however, the quality of specimens is poor for the G-banding analysis due to spontaneous discharge, array-CGH supplemented with STR analysis can be a powerful technique for cytogenetic evaluation of miscarriage, since STR analysis allows for the detection of triploidy and part of tetraploidy as well as maternal contamination.

In recent years, single-nucleotide polymorphism microarray (SNP array) or next generation sequencing has been also introduced in cytogenetic analysis of POC specimens (Lathi et al. 2012; Bug et al. 2014; Kooper et al. 2014; Levy et al. 2014; Maslow et al. 2015; Liu et al. 2015; Shen et al. 2016). SNP array not only overcomes several shortcomings of traditional karyotyping but also has the advantage of detecting ploidy status except tetraploidy derived from cytokinesis failure, compared with array-CGH (Lathi et al. 2012). In addition, if parental DNAs are simultaneously analyzed, this technique can identify parental source of aneuploidies, uniparental disomy (UPD) and maternal contamination in a single methodology (Lathi et al. 2012; Bug et al. 2014). The recent report demonstrated that over half of the normal female results in miscarriage specimens were resulted from maternal cell contamination by the confirmatory analysis using SNP array (Lathi et al. 2014). On the other hand, submicroscopic imbalances additionally detected by high-resolution array are reported to include variants of unknown significance (Dhillon et al. 2013; Bug et al. 2014; Levy et al. 2014), and even common submicroscopic imbalances are of dubious value whether it contributes to the cause of miscarriage. Currently available cytogenetic tests capable of detecting aneuploidies and subtelomeric imbalances may be sufficient in daily clinical practice for RPL. However, to further understand the cytogenetic mechanisms underlying early fetal development and to potentially open up new avenues for the management of RPL, high resolution genome-wide assay(s) will be needed (Dhillon et al. 2013; Viaggi et al. 2013; Kooper et al. 2014; Wen et al. 2015; Zhou et al. 2016).

## Conclusions

In this study, we analyzed 15 spontaneously discharged POCs by array-CGH and successfully obtained cytogenetic results from all specimens. The additional STR analysis for normal female results were effective to evaluate the contamination of maternal DNA. We believe that array-CGH supplemented with STR analysis could be a greatly beneficial means to analyze spontaneously discharged POC specimens, especially for women with RPL who desire expectant management and/or a thorough investigation on the cause for RPL.

## Methods

Spontaneously discharged POCs were collected from women with missed abortion who desired expectant management. In this study, the specimens were restricted to POCs spontaneously discharged at home and collected by patients. POCs discharged at hospitals were excluded. All tissue samples were grossly examined, and part of chorionic villi were separated (the remainder was used for pathologic examination) and washed with normal saline solution two or three times, removing maternal blood clots, bloody tissues and decidua remains. The cleaned tissues were frozen and stored at −20°C until use. Genomic DNA was extracted using a standard kit.

Array-CGH was performed using three versions of targeting BAC-based array, designated Genome Disorder Array (GDA); GDA version 2, GDA version 3 and GD-700, which included 550, 660 and 712 BACs, respectively. The amount of DNA used for these analyses was 1.5, 1.5 and 4.5 μg, respectively. These arrays cover causative lesions of approximately 30 genetic diseases and subtelomeric regions of all chromosomes except for 13p, 14p, 15p, 21p and 22p (Hayashi et al. 2011). By using several array platforms including those GDAs, cryptic chromosome aberrations were comprehensively screened in 645 Japanese patients with intellectual disabilities and/or multiple congenital anomalies of unknown etiology (Hayashi et al. 2011; Uehara et al. 2016). In this study, normal male DNA was used as control and gender of POC was estimated by X/Y signal patterns. Thresholds for copy-number gain and loss were set at 1.25 and 0.75, respectively. In cases of normal female results without copy number changes, maternal DNA contamination was additionally evaluated by comparison of STR patterns with maternal peripheral blood. STR analysis was performed using GenomeLab Human STR Primer Set (Beckman Coulter, Tokyo, Japan) that included 12 STR markers. The protocol of this study was approved by the institutional ethical committee. Informed consent was obtained from all participants for DNA extraction and DNA-based analysis.

## Additional files

10.1186/s40064-016-2594-6 GDA results (Cases 1–15). Cases 1–6 were analyzed by GDA Ver. 2 (550BACs), Cases 7–9 by GDA Ver. 3 (660BACs) and Cases 10–15 by GD-700 (712BACs). The *x-axis* indicates array spots of BAC clones ordered from chromosomes 1–22, X and Y. The *y-axis* shows the fluorescence ratio of differently labeled sample/control DNA. Normal male DNA was used as control in this study. Thresholds for copy-number gain and loss were defined at 1.25 and 0.75, respectively.
10.1186/s40064-016-2594-6 GDA results of the parents of Case 2. (A) Mother, (B) Father. The parents’ DNA were extracted from their peripheral blood and analyzed by GDA Ver. 2 (550BACs). Normal female and male DNA were used as control, respectively. Both of their GDA results had no abnormal findings.

## Abbreviations

POC: products of conception
RPL: recurrent pregnancy loss
array-CGH: array-based comparative genomic hybridization
STR: short tandem repeat
D&C: dilation and curettage
BAC: bacterial artificial chromosome
GDA: Genome Disorder Array
FISH: fluorescence in situ hybridization
SNP: single-nucleotide polymorphism
