# Supplementary material for: Cytogenetic analysis of spontaneously discharged products of conception by array-based comparative genomic hybridization
Source: Springerplus. 2016 Jun 24;5(1):874. doi: 10.1186/s40064-016-2594-6 (PMC4920787; doi:10.1186/s40064-016-2594-6)
Supplement: Supplementary file 1 — 10.1186/s40064-016-2594-6 GDA results (Cases 1–15). Cases 1–6 were analyzed by GDA Ver. 2 (550BACs), Cases 7–9 by GDA Ver. 3 (660BACs) and Cases 10–15 by GD-700 (712BACs). The x-axis indicates array spots of BAC clones ordered from chromosomes 1–22, X and Y. The y-axis shows the fluorescence ratio of differently labeled sample/control DNA. Normal male DNA was used as control in this study. Thresholds for copy-number gain and loss were defined at 1.25 and 0.75, respectively. [file 40064_2016_2594_MOESM1_ESM.pptx]

## Slide 1
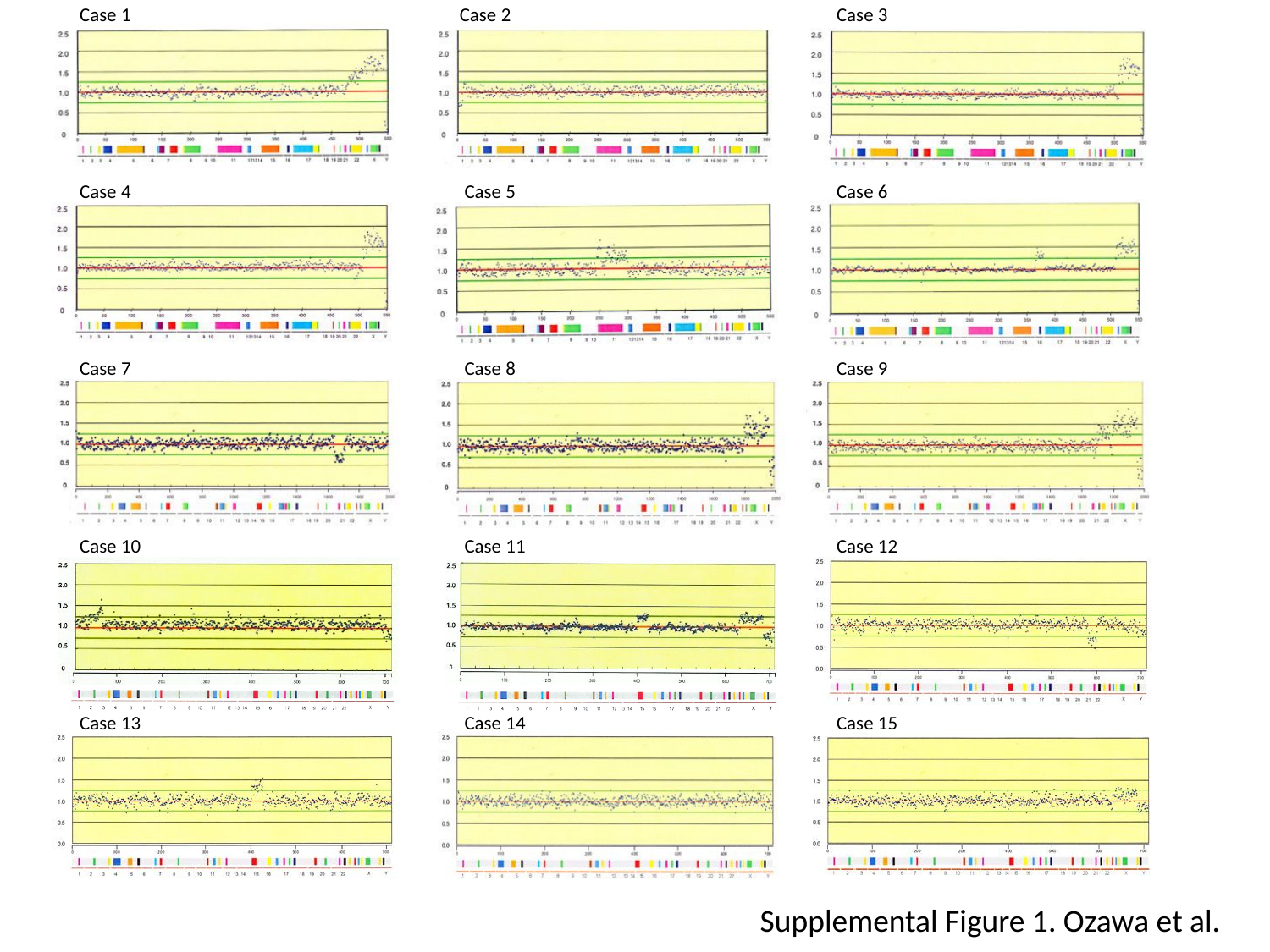

Case 1
Case 2
Case 3
Case 4
Case 5
Case 6
Case 7
Case 8
Case 9
Case 10
Case 11
Case 12
Case 13
Case 14
Case 15
Supplemental Figure 1. Ozawa et al.
