# Supplementary figures and images for: Cytogenetic analysis of spontaneously discharged products of conception by array-based comparative genomic hybridization
Source: Springerplus. 2016 Jun 24;5(1):874. doi: 10.1186/s40064-016-2594-6 (PMC4920787; doi:10.1186/s40064-016-2594-6)

## Slide 1
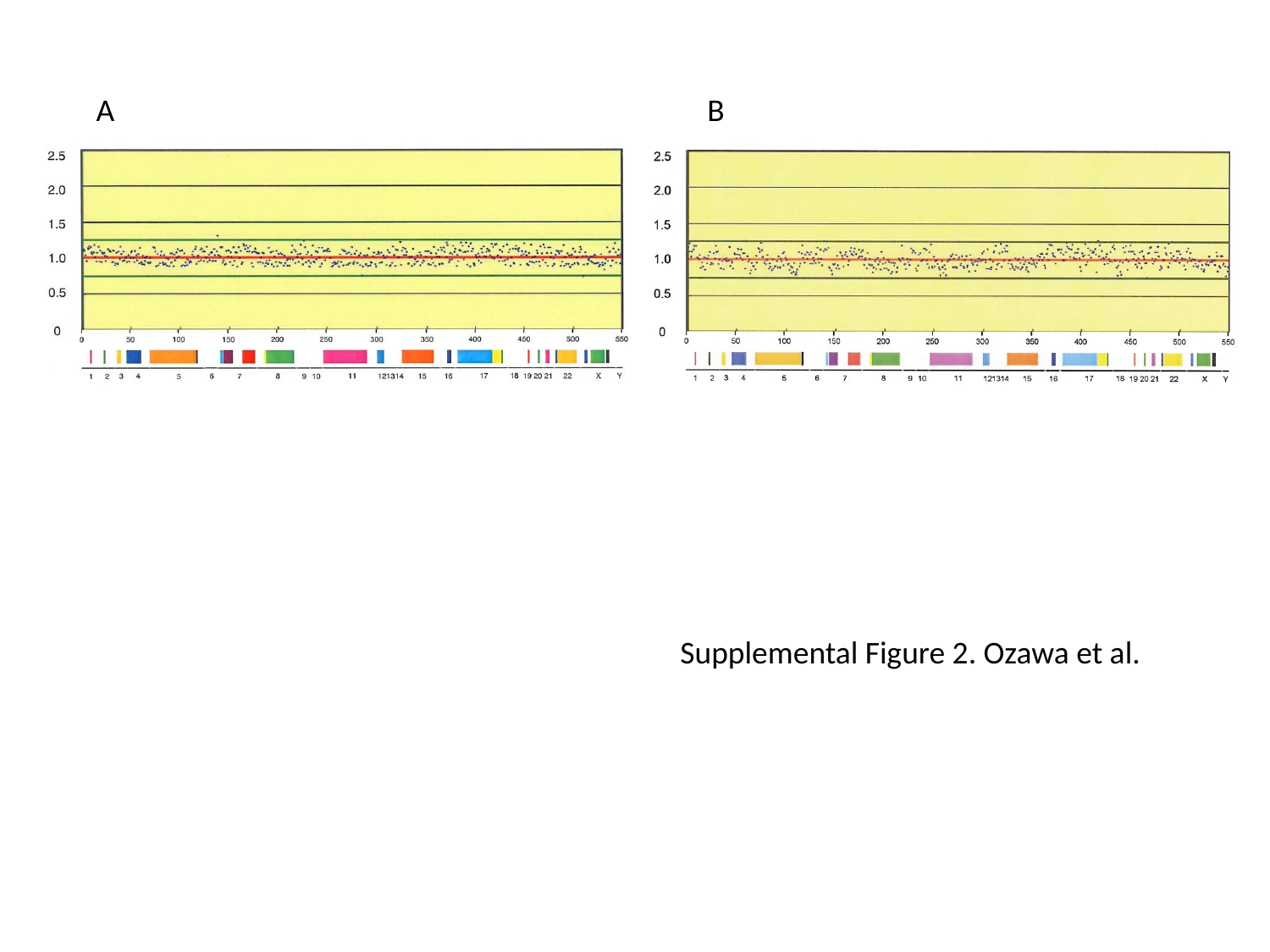

A
B
Supplemental Figure 2. Ozawa et al.

Supplement: Supplementary file 2 — 10.1186/s40064-016-2594-6 GDA results of the parents of Case 2. (A) Mother, (B) Father. The parents’ DNA were extracted from their peripheral blood and analyzed by GDA Ver. 2 (550BACs). Normal female and male DNA were used as control, respectively. Both of their GDA results had no abnormal findings. [file 40064_2016_2594_MOESM2_ESM.pptx]
